# Antisense inhibition of RNA polymerase α subunit of *Clostridioides difficile*

**DOI:** 10.1128/spectrum.01755-23

**Published:** 2023-09-29

**Authors:** Rusha Pal, Mohamed N. Seleem

**Affiliations:** 1 Department of Biomedical Sciences and Pathobiology, Virginia-Maryland College of Veterinary Medicine, Virginia Polytechnic Institute and State University, Blacksburg, Virginia, USA; 2 Center for Emerging Zoonotic and Arthropod-borne Pathogens, Virginia Polytechnic Institute and State University, Blacksburg, Virginia, USA; Universite Paris-Saclay, Gif-sur-Yvette, France

**Keywords:** *C. difficile*, peptide nucleic acids, *rpoA*, virulence factors, toxins, spores, gut microflora

## Abstract

*Clostridioides difficile,* the causative agent of antibiotic-associated diarrhea and pseudomembranous colitis, has emerged as a major enteric pathogen in recent years. Antibiotic treatment perturbs the gut microbiome homeostasis, which facilitates the colonization and proliferation of the pathogen in the host intestine. Paradoxically, the clinical repertoire for *C. difficile* infection includes the antibiotics vancomycin and/or fidaxomicin. The current therapies do not address the perturbed gut microbiome, which supports the recurrence of infection after cessation of antibiotic therapy. Peptide nucleic acids (PNAs) are novel alternatives to traditional antimicrobial therapy capable of forming strong and stable complexes with RNA and DNA, thus permitting targeted inhibition of specific genes. Here, we report a novel PNA that can target the RNA polymerase α subunit (*rpoA*) in *C. difficile*. The designed anti-*rpoA* construct inhibited clinical isolates of *C. difficile* (minimum inhibitory concentration values ranged between 4 and 8 µM) and exhibited bactericidal activity. Furthermore, silencing of the *rpoA* gene suppressed the expression of genes that encode virulence factors [toxin A (*tcdA*), toxin B (*tcdB*)] in *C. difficile*, and the gene that encodes the transcription factor stage 0 sporulation protein (*spoOA*). Interestingly, the efficacy of the designed PNA conjugate remained unaffected even when tested at different pH levels and against a high inoculum of the pathogen. The *rpoA*-TAT conjugate was very specific against *C. difficile* and did not inhibit members of the beneficial gut microflora. Taken altogether, our study confirms that the *rpoA* gene can be a promising narrow-spectrum therapeutic target to curb *C. difficile* infection.

**IMPORTANCE:**

The widespread use of antibiotics can destroy beneficial intestinal microflora, opening the door for spores of *Clostridioides difficile* to run rampant in the digestive system, causing life-threatening diarrhea. Alternative approaches to target this deadly pathogen are urgently needed. We utilized targeted therapeutics called peptide nucleic acids (PNAs) to inhibit gene expression in *C. difficile*. Inhibition of the RNA polymerase α subunit gene (*rpo*A) by PNA was found to be lethal for *C. difficile* and could also disarm its virulence factors. Additionally, antisense inhibition of the *C. difficile rpo*A gene did not impact healthy microflora. We also propose a novel approach to manipulate gene expression in *C. difficile* without the need for established genetic tools.

## INTRODUCTION

*Clostridioides difficile* (formerly known as *Clostridium difficile*) is an anaerobic, Gram-positive, toxin-producing, and spore-forming intestinal bacterium capable of infecting and causing lethal diarrhea in humans. The United States (US) Centers for Disease Control and Prevention (CDC) has classified *C. difficile* as an urgent threat and the most common cause of healthcare-associated infections; *C. difficile* infection (CDI) resulted in 12,800 deaths in the US in 2017 (1).

The use of broad-spectrum antibiotics perturbs the indigenous intestinal microbial community, which primes the human gut to become prone to *C. difficile* colonization and disease manifestation (2). Paradoxically, the treatment repertoire for *C. difficile* infection includes antibiotics like vancomycin and fidaxomicin (3, 4). Formerly a first-line treatment agent for CDI, metronidazole is now recommended for use only in patients who have limited access to vancomycin and fidaxomicin (3). Though associated with a high initial cure rate (>80%), 15–30% of patients treated with vancomycin and fidaxomicin experience a first episode of *C. difficile* recurrence with a subsequent increase in the risk of further CDI recurrence (5). An alternative approach to antibiotics that has been touted as the most effective method to treat refractory CDI is fecal microbiota transplantation (FMT) from healthy donors (6). Randomized controlled trials for FMT have reported over 90% efficacy for the treatment of CDI (7). However, investigational FMT recently led to extended-spectrum β-lactamase producing *Escherichia coli* bacteremia in two individuals, with one individual succumbing to the infection (8). Additional concerns with FMT include the lack of standard protocols and the unknown adverse consequences of long-term treatment (9). The US Food and Drug Administration has recently approved an oral microbiota therapy by Seres Therapeutics. SER-109 is aimed at preventing recurrent CDI. While SER-109 is intended to tackle CDI recurrence, antibiotics still constitute the standard-of-care therapy (10). The drawbacks associated with the current therapeutic arsenal highlight the need for the development of novel treatment strategies for the treatment of CDI and the prevention of CDI recurrence.

An alternative strategy for developing small molecules can be the exploitation of the antisense principle for specific gene inhibition in bacterial pathogens. The design of peptide nucleic acid (PNA) oligomers represents one such antisense tool that harbors the potential to effectively combat hard-to-treat infectious agents. PNAs are polynucleotide analogs that can hybridize to complementary RNA and DNA in a sequence-dependent manner (11 – 14). PNAs differ from naïve DNA or RNA molecules in that the sugar-phosphate backbone in nucleic acids is replaced with a noncyclic peptide-like backbone in PNAs. This confers the PNA with increased hybridization affinity, high chemical stability at different pHs and temperatures, and makes the PNA resistant to serum proteases and nucleases (15, 16). Additionally, PNAs are known to be non-toxic *in vivo* as they could successfully reduce the bacterial load of *Staphylococcus aureus* in a murine infection model without demonstrating any toxic effect on mice (15). However, the success of such nucleic acid-based strategies in the field of drug discovery/development has been limited due to insufficient delivery technologies (17). Hence, researchers have explored the merits of a novel delivery strategy that involves conjugating PNAs to cell penetrating peptides (CPPs) to aid/enhance the cellular uptake of carrier PNA-CPP conjugates (17).

In this study, we report the use of a PNA oligomer as an antisense probe against the RNA polymerase α subunit (*rpoA*) of *C. difficile*. We evaluated the antibacterial ability of our designed peptide-PNA conjugate to inhibit the growth and virulence factors of *C. difficile* clinical isolates. Interestingly, *rpoA*-TAT retained its activity against a high inoculum of *C. difficile* and inhibited the pathogen even at high pH. To verify the mechanism of inhibition, we analyzed the expression of the *rpoA* gene, which revealed that our PNA conjugate downregulated the expression of *rpoA*. Additionally, our PNA conjugate inhibited the expression of multiple genes that encode virulence factors of *C. difficile*. The *rpoA*-TAT conjugate also did not inhibit the growth of multiple commensal microflora strains. The promising anticlostridial efficacy *in vitro* highlights that peptide-PNA conjugates provide an alternative treatment approach to combat *C. difficile* and its virulence traits.

## RESULTS

### PNA target site selection

The sequence of the *rpoA* 5′ terminal region of several *C. difficile* isolates was aligned using the Basic Local Alignment Search Tool. Based on the analysis, the antisense oligonucleotide was designed to target a region of the *rpoA* gene that is conserved across isolates of *C. difficile* (Table S1). The target site, which includes the start codon and nine nucleotides upstream, is accessible for ribosome assembly and is sensitive to antisense inhibition, as demonstrated previously (18 – 20). The antisense oligonucleotide was covalently conjugated to five different CPPs (Table 1).

**TABLE 1:** Antisense constructs used in this study

PNA constructs/CPP^*a*^	Sequence
TAT-*rpoA*	GRKKKRRQRRRYK-O-CATGGACAAAAC-NH_2_
(RXR)_4_XB-*rpoA*	RXRRXRRXRRXRXB-O-CATGGACAAAAC-NH_2_
(RFR)_4_XB-*rpoA*	RFRRFRRFRRFRXB-O-CATGGACAAAAC-NH_2_
K8-*rpoA*	KKKKKKKK-O-CATGGACAAAAC-NH_2_
(KFF)_3_K-*rpoA*	KFFKFFKFFK-O-CATGGACAAAAC-NH_2_
TAT-*rpoA* mismatch	GRKKKRRQRRRYK-O-CATACAATTCTC-NH 2
TAT	GRKKKRRQRRRYK

^*a*^ CPP, cell penetrating peptide; PNA, peptide nucleic acid.

### *In vitro* susceptibility of *C. difficile* to the PNA constructs

The susceptibility of *C. difficile* ATCC 630 to the PNA conjugates was evaluated *in vitro*. Three PNA conjugates, TAT-*rpoA*, (RXR)_4_XB -*rpoA*, and (RFR)_4_XB-*rpoA*, inhibited growth of *C. difficile* ATCC 630 at a concentration of 8 µM (Table 2). Two of the PNA conjugates, K8-*rpoA* and (KFF)_3_K-*rpoA,* inhibited the growth of *C. difficile* 630 at a concentration of 32 µM. All the PNA constructs were bactericidal. The MIC of the TAT CPP alone was also evaluated, but up to a concentration of 64 µM, the TAT CPP failed to inhibit the growth of *C. difficile* 630 (Table 2). The TAT-*rpoA* mismatch did not exhibit any antibacterial activity up to a concentration of 32 µM (Table 2).

**TABLE 2:** Impact of CPPs on the antisense effect of the *rpoA* PNA construct^*a*^

PNA	*C. difficile* ATCC 630	
	MIC (µM)	MBC (µM)
TAT-*rpoA*	8	8
(RXR)_4_XB -*rpoA*	8	8
(RFR)_4_XB -*rpoA*	8	8
K8-*rpoA*	32	32
(KFF)_3_K-*rpoA*	32	32
TAT- *rpoA* mismatch	>32	>32
HIV-TAT	>64	NT

^*a*^ ATCC, American Type Culture Collection; CPP, cell penetrating peptide; MBC, minimum bactericidal concentration; MIC, minimum inhibitory concentration; NT, not tested; PNA, peptide nucleic acid.

### *In vitro* susceptibility of clinical isolates of *C. difficile* to the *rpoA*-TAT conjugate and free TAT

The susceptibility of *C. difficile* clinical isolates to the *rpoA*-TAT conjugate *in vitro* was determined. As shown in Table 3, *rpoA*-TAT displayed potent anticlostridial activity with MIC values that ranged between 4 and 8 µM against clinical isolates of *C. difficile*. The TAT-*rpoA* mismatch did not exhibit any antibacterial activity up to a concentration of 32 µM. The antisense PNA *rpoA*-TAT also demonstrated bactericidal activity against *C. difficile* isolates similar to the standard-of-care antibiotics vancomycin and fidaxomicin.

**TABLE 3:** MIC and MBC of *rpoA*-TAT against clinical isolates of *C. difficile^a^*

Strains/clinical isolates	*rpoA*-TAT		*rpoA*-TAT mismatch		Vancomycin		Fidaxomicin	
	MIC (µM)	MBC (µM)	MIC (µM	MBC (µM)	MIC (µM)	MBC (µM)	MIC (µM)	MBC (µM)
*C. difficile* ATCC 630	8	8	>32	>32	2	2	0.5	0.5
*C. difficile* ATCC BAA 1870	8	8	>32	>32	4	4	1	1
*C. difficile* ATCC 43255	8	8	>32	>32	4	4	0.5	0.5
*C. difficile* 1071	8	8	NT	NT	2	2	1	1
*C. difficile* 1079	8	8	NT	NT	2	2	2	2
*C. difficile* 1082	4	4	NT	NT	2	2	2	2

^*a*^ ATCC, American Type Culture Collection; MBC, minimum bactericidal concentration; MIC, minimum inhibitory concentration; NT, not tested; PNA, peptide nucleic acid.

### The PNA-TAT conjugate displays rapid bactericidal activity

**Fig 1:**
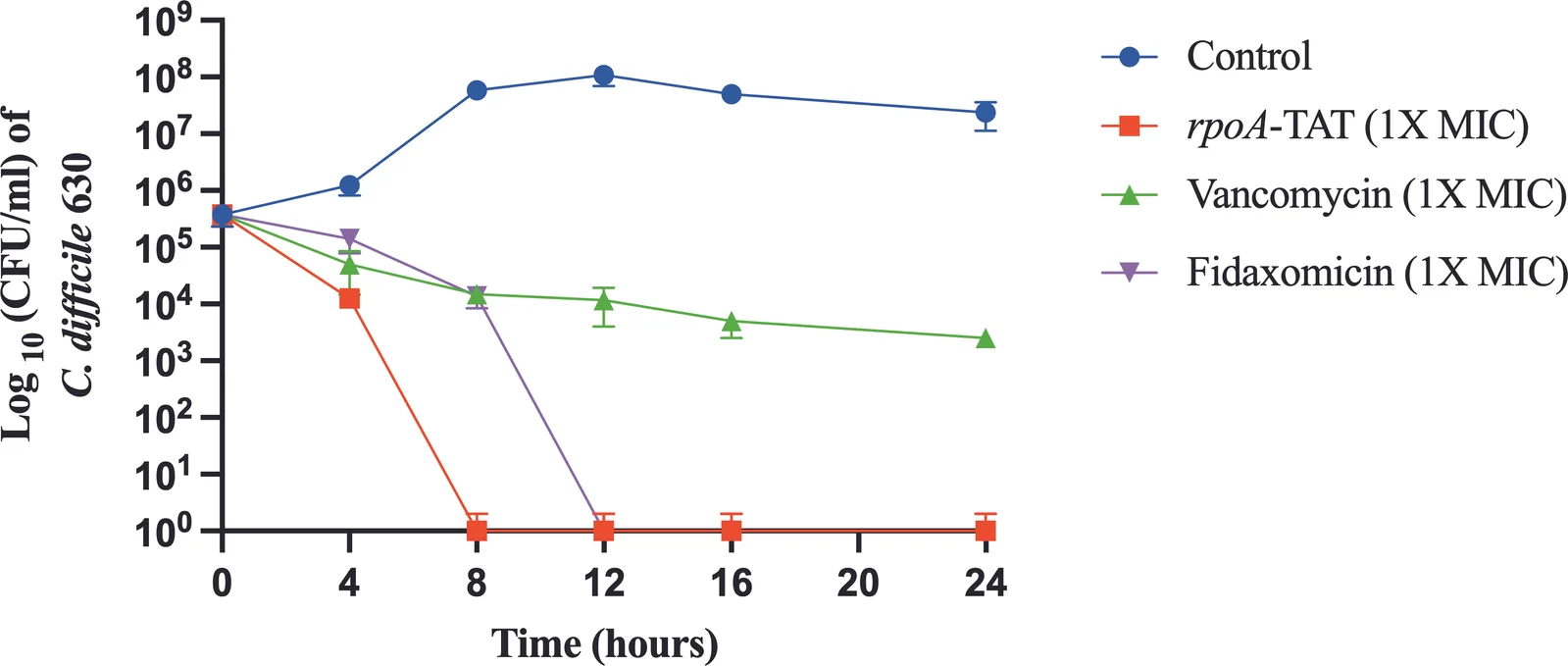
Killing kinetics of *rpoA-*TAT, vancomycin, and fidaxomicin against *C. difficile* 630. The effect of each treatment group was evaluated in triplicate. The error bars represent standard deviation values.

The killing kinetics of the *rpoA*-TAT conjugate, vancomycin, and fidaxomicin were evaluated against *C. difficile* 630. The inoculum size at time zero for the time-kill assay was ~5 × 10^5^ CFU/mL. As shown in Fig. 1, *rpoA*-TAT eradicated bacteria within 8 hours of incubation (below the limit of detection, 250 CFU/mL). Fidaxomicin eradicated the bacteria within 12 hours of incubation, whereas vancomycin reduced the bacterial burden by ~1.6 log_10_ CFU/mL within 24 hours of incubation.

### Effect of high bacterial inoculum on PNA-TAT activity

The effect of a high inoculum of *C. difficile* 630 on the antibacterial activity of the *rpoA*-TAT conjugate was determined. The PNA-TAT conjugate cleared a high inoculum count (~5 × 10^6^ and ~5 × 10^7^ cells) of *C. difficile* ATCC 630. The PNA-TAT conjugate was as effective as fidaxomicin in clearing the pathogen, whereas vancomycin demonstrated reduced antibacterial activity in the presence of a high inoculum of *C. difficile* (Table 4).

**TABLE 4:** Effect of high inoculum of *C. difficile* 630 on the activity of *rpoA*-TAT and control antibiotics^*a*^

1× MIC (8 hours)	5 × 10^6^		5 × 10^7^	
	Log CFU	Log reduction	Log CFU	Log reduction
No treatment	6.37	None	7.77	None
Vancomycin	5.37	1	7.03	0.73
Fidaxomicin	0	6.37	0	7.77
*rpoA*-TAT	0	6.37	0	7.77

^*a*^ CFU, colony-forming units; MIC, minimum inhibitory concentration.

### Effect of pH on PNA-TAT activity

The susceptibility of *C. difficile* (strains ATCC 630 and ATCC BAA 1870) to *rpoA*-TAT and control antibiotics over a pH range of 6 to 8 was evaluated using the broth microdilution method. The MIC values for vancomycin were found to increase with increasing pH; the MIC value of vancomycin increased by two- to threefold (at pH 8) compared to the MIC value at the lowest pH (pH 6). The MIC values for fidaxomicin at pH 8 increased by onefold for *C. difficile* ATCC 630 and remained unchanged even at a higher pH for *C. difficile* ATCC BAA 1870. Interestingly, the MIC values for *rpoA*-TAT were found to remain constant over the pH range of 6–8 for both the isolates of *C. difficile* tested (Table 5).

**TABLE 5:** Effect of pH on the anticlostridial activity of *rpoA*-TAT and control antibiotics against *C. difficile* 630 and *C. difficile* 1870^*a*^

PNA/control antibiotics	pH 6				pH 7				pH 8			
	*C. difficile* ATCC 630		*C. difficile* ATCC BAA 1870		*C. difficile* ATCC 630		*C. difficile* ATCC BAA 1870		*C. difficile* ATCC 630		*C. difficile* ATCC BAA 1870	
	MIC (µM)	MBC (µM)	MIC (µM)	MBC (µM)	MIC (µM)	MBC (µM)	MIC (µM)	MBC (µM)	MIC (µM)	MBC (µM)	MIC (µM)	MBC (µM)
*rpoA*-TAT	8	8	8	8	8	8	8	8	8	8	4	4
Vancomycin	2	2	4	4	4	4	4	8	8	16	8	16
Fidaxomicin	0.5	1	1	2	0.5	1	1	2	1	4	1	4

^*a*^ ATCC, American Type Culture Collection; MBC, minimum bactericidal concentration; MIC, minimum inhibitory concentration; PNA, peptide nucleic acid.

### The PNA-TAT conjugate exhibits dose-dependent inhibition of *rpoA* gene expression and the expression of genes encoding key virulence factors (*tcdA*, *tcdB*, and *spoOA*) in *C. difficile*

**Fig 2:**
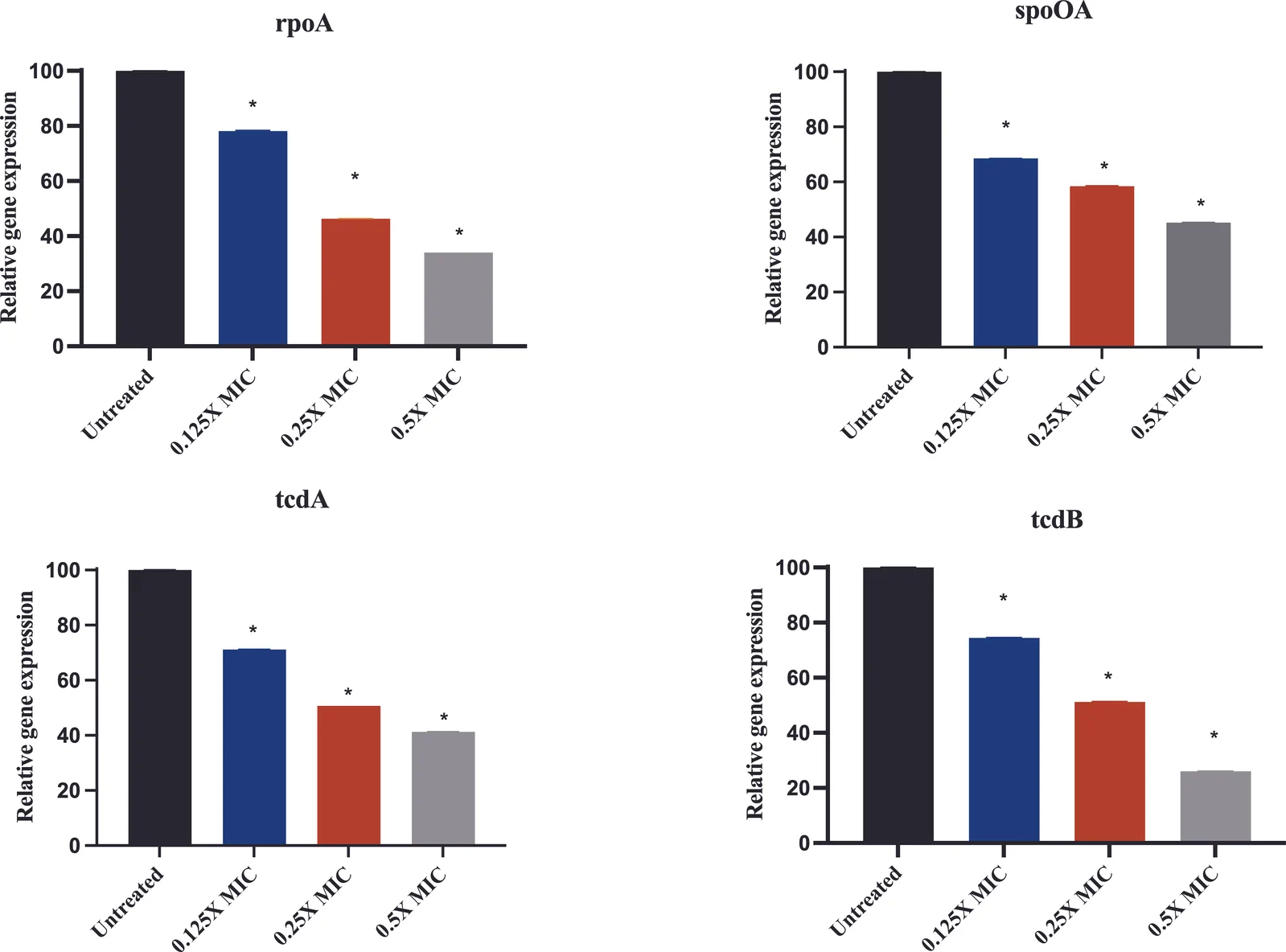
Dose-dependent inhibition of *C. difficile* ATCC 630 *rpoA*, *spoOA*, *tcdA*, and *tcdB* genes after incubation with the *rpoA*-TAT conjugate. An overnight culture of *C. difficile* 630 was diluted and incubated with 0.125× MIC, 0.25× MIC, and/or 0.5× MIC of *rpoA*-TAT for 12 hours. Water served as the untreated group. Total RNA was extracted, and the level of gene expression was determined using qPCR. Topoisomerase (*tpi*) was used as an internal control. An asterisk (*) denotes a statistically significant difference in gene expression for the untreated control compared to the treated controls (*P* < 0.001) using an unpaired Student’s *t*-test.

The antisense effect of the PNA-TAT construct was evaluated using RT-PCR. As depicted in Fig. 2, *rpoA*-TAT reduced *rpoA* gene expression by 34% at 0.125× MIC, 46.3% at 0.25× MIC, and 78.2% at 0.5× MIC. Furthermore, suppression of *rpoA* gene expression led to significant downregulation of genes that encode virulence factors (toxins and spores) for *C. difficile*. The *rpoA*-TAT construct reduced toxin A (*tcdA*) gene expression by 41.3% at 0.125× MIC, 50.7% at 0.25× MIC, and 71.2% at 0.5× MIC, respectively. The *rpoA*-TAT construct reduced toxin B (*tcdB*) gene expression by 26% at 0.125× MIC, 51.2% at 0.25× MIC, and 74.5% at 0.5× MIC, respectively. Furthermore, the *rpoA*-TAT construct reduced expression of the *spoOA* gene (encoding for the transcription factor stage 0 sporulation protein) by 45.2% at 0.125× MIC, 58.4% at 0.25× MIC, and 68.5% at 0.5× MIC.

### The PNA-TAT conjugate inhibits toxin A and toxin B production by *C. difficile* 630

**Fig 3:**
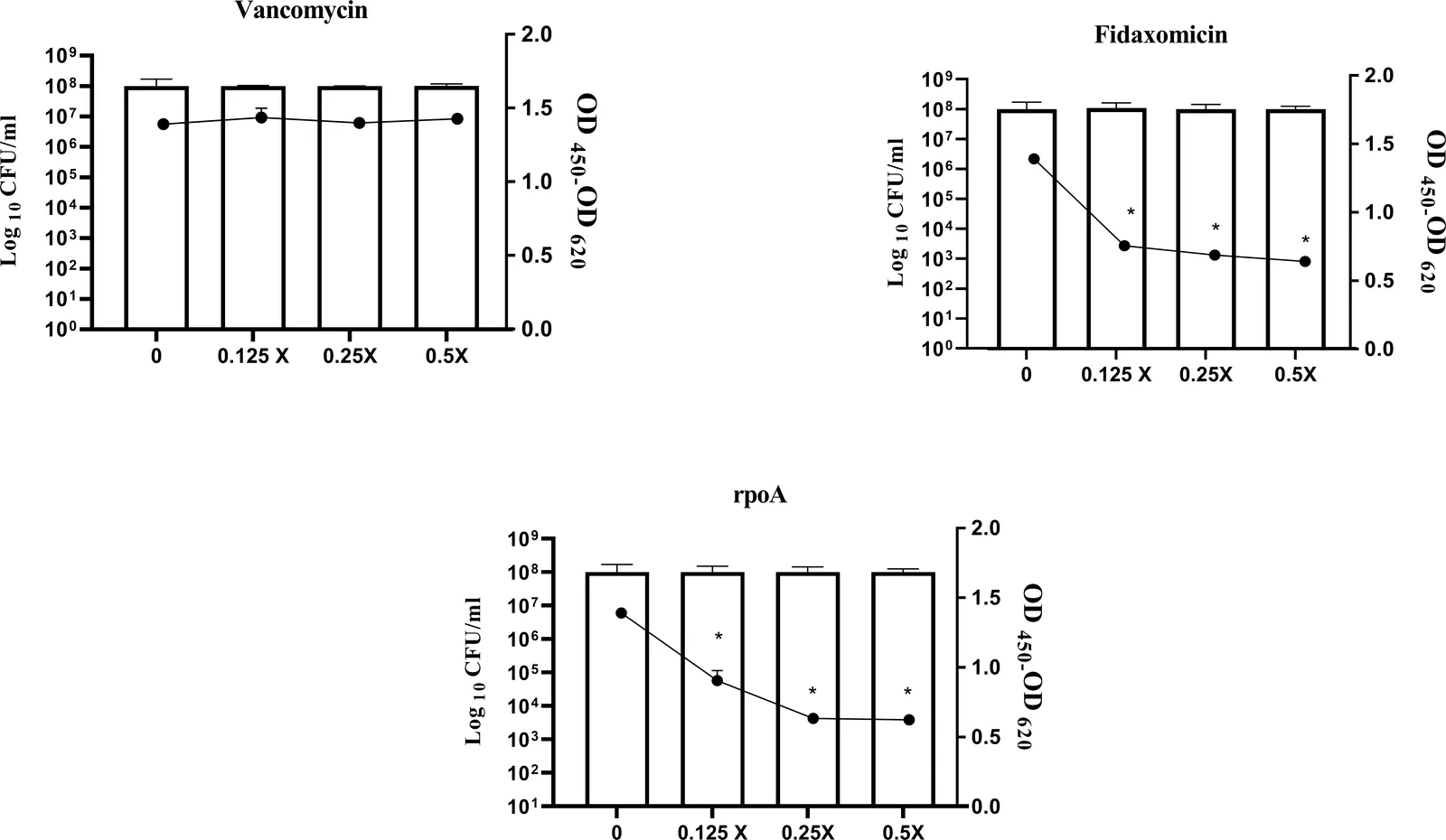
*C. difficile* toxin inhibition by the *rpoA*-TAT conjugate and control antibiotics. *C. difficile* 630 was incubated with subinhibitory concentrations of the *rpoA*-TAT conjugate, fidaxomicin, or vancomycin for 8 hours. The number of viable cells (log_10_ CFU/mL, bars) in each treatment group was determined. An ELISA kit was used to evaluate the presence of toxin in each supernatant (OD_450_–OD_620_, lines). Each treatment was done in triplicate, and the data represent the mean and standard deviation (depicted by the error bars) values. An asterisk (*) denotes a statistically significant difference in the toxin content of the supernatant between the untreated group and each treatment group analyzed using an ordinary one-way ANOVA.

As shown in Fig. 3, when *C. difficile* 630 was incubated with sub-inhibitory concentrations of the PNA-TAT conjugate (0.125× MIC, 0.25× MIC, and 0.5× MIC), a reduction in toxin production was observed without affecting the viability of the pathogen. The *rpoA*-TAT conjugate reduced *C. difficile* toxin production by 34.9% at 0.125× MIC, 54.3% at 0.25 × MIC, and 55.2% at 0.5× MIC, respectively. As for the control antibiotics, vancomycin did not inhibit toxin production by *C. difficile,* while fidaxomicin exhibited a dose-dependent inhibition of toxin production by *C. difficile* (45.7% inhibition at 0.125× MIC, 50.6% inhibition at 0.25× MIC, and 54% inhibition at 0.5× MIC, respectively).

### The PNA-TAT conjugate inhibits spore formation by *C. difficile* ATCC 630

**Fig 4:**
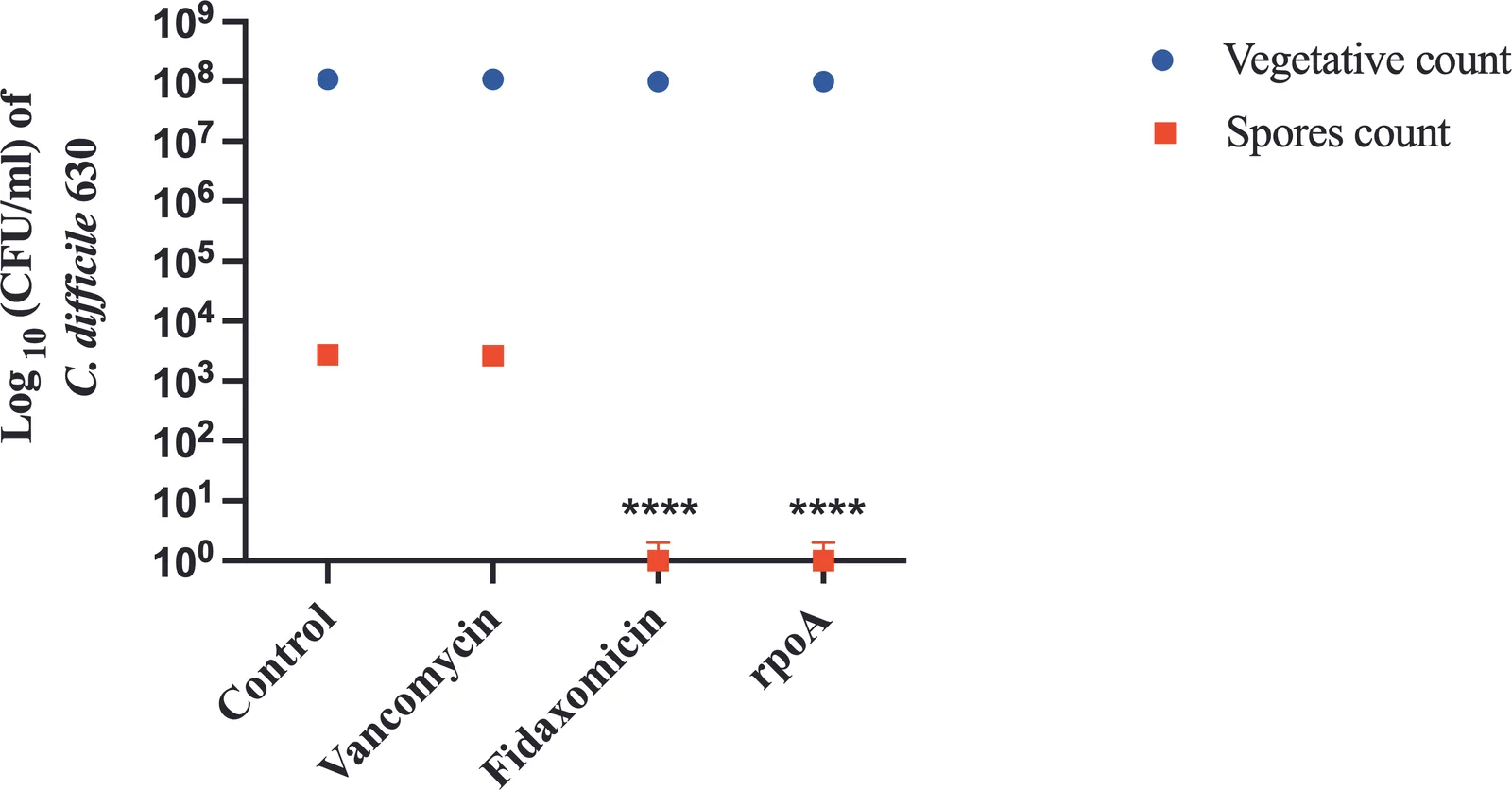
Spore inhibition by the *rpoA*-TAT conjugate against *C. difficile* ATCC 630 along with the control antibiotics vancomycin and fidaxomicin. A sub-inhibitory concentration (0.5× MIC) of each treatment was incubated with bacteria for 12 hours before the total bacterial count and spore count were determined. Water served as the negative control. The error bars represent the standard deviation values for triplicate samples of each treatment. Asterisks (****) denote statistically significant differences between the vegetative count (blue circle) and the spore count (red squares) for each treatment analyzed via an unpaired Student’s *t*-test.

Treatment of *C. difficile* ATCC 630 with a sub-inhibitory concentration of the *rpoA*-TAT conjugate (0.5× MIC) inhibited spore formation (Fig. 4) without affecting the viability of the bacterial cells. Both the *rpoA*-TAT conjugate and fidaxomicin exhibited a 3.44-log reduction (99.9% reduction) in the spore count by *C. difficile* compared to the negative control (no treatment). Vancomycin did not inhibit spore formation by *C. difficile*.

### The PNA-TAT conjugate did not inhibit the growth of gut microflora

The PNA-TAT construct did not inhibit the growth of the representative gut microflora strains (*Lactobacillus* sp., *Bacteroides* sp., and *Bifidobacterium* sp.) at a concentration of 32 µM. In contrast, vancomycin inhibited the growth *of L. gasseri* (MIC = 1 µM) and *B. longum* (MIC = 2 µM). Fidaxomicin inhibited the growth of *B. longum* (MIC = 0.125 µM)(Table 6).

**TABLE 6:** MIC values for the *rpoA*-TAT conjugate and control antibiotics against gut microflora strains

PNA/Control antibiotics	MIC^*a*^ (μM)		
	*rpoA*-TAT	Vancomycin	Fidaxomicin
*Lactobacillus brevis* ATCC 14869	>32	>64	>2
*Lactobacillus gasseri* ATCC 19992	>32	1	>2
*Lactobacillus rhamnosus* ATCC 53103	>32	>64	>2
*Bacteroides fragilis* HM 714	>32	64	>2
*Bifidobacterium longum* subsp. *longum* HM 846	>32	2	0.125

^*a*^ MIC, minimum inhibitory concentration; PNA, peptide nucleic acid.

## DISCUSSION

The enteric pathogen *C. difficile* is a global public health threat that can cause debilitating gastrointestinal symptoms. The use of antibiotics paves the way for CDI by altering the gut microflora, thus placing certain populations (such as patients with prolonged hospitalizations and elderly people) at high risk for infection. Rather incongruously, the glycopeptide antibiotic vancomycin and the macrolide antibiotic fidaxomicin are used to treat CDI (3, 4). Vancomycin, although effective at killing *C. difficile* vegetative cells, causes further damage to the gut microflora, thereby resulting in disease recurrence (21). Fidaxomicin, a novel macrolide, has a narrower spectrum of activity compared to vancomycin and has a superior sustained clinical response. However, the rate of CDI recurrence has been found to be similar for patients infected with *C. difficile* ribotype 027 strains who are treated with either vancomycin or fidaxomicin (22, 23). Hence, an alternative approach to traditional antibiotics that can spare the gut microbiome and target the virulence factors of the pathogen is highly desirable.

Antisense gene inhibition occurs naturally in bacteria (24). Building on this, an antisense approach has been developed that can inhibit bacterial gene expression along with cell growth (25). These antisense PNAs have a pseudo-peptide backbone that offers increased hybridization to DNA and RNA and provides biological stability, thus raising the prospects of PNAs as novel antibacterial agents. However, because PNAs are large and hydrophilic molecules, they cannot efficiently enter bacterial cells. Hence, the PNA design must be modified by linking the antisense PNAs to a cell wall permeabilizing agent (25). In this study, we designed such a peptide-PNA conjugate targeting the *rpoA* gene in *C. difficile*.

Among the five PNA conjugates designed to target the *rpoA* gene, we identified three PNA conjugates [TAT-*rpoA*, (RXR)_4_XB -*rpoA*, and (RFR)_4_XB-*rpoA*] that exhibited strong bactericidal activity against *C. difficile*. We pursued further experiments with TAT conjugated to the anti-*rpoA* PNA because a previous study found that TAT can enhance the antisense effect of anti-*gyrA* in *Streptococcus pyogenes* (26). Additionally, TAT-fused peptides are increasingly under clinical development (27 – 29). Our *rpoA*-TAT construct was found to be effective in inhibiting the growth of clinical isolates of *C. difficile*. Furthermore, the *rpoA*-TAT conjugate cleared bacterial burden at a faster pace than the standard-of-care antibiotic vancomycin; this could be clinically important because the PNA-conjugate can rapidly resolve infection (30). The antimicrobial activity of any compound can be influenced by bacterial density. Hence, the activity of the PNA construct was investigated against a high inoculum of *C. difficile*. Interestingly, the PNA constructs retained potent activity against a high inoculum of *C. difficile*. The PNA construct also retained its efficacy at basic pH, indicating that the peptide-PNA conjugate can be expected to harbor full anticlostridial activity at the physiological pH of the human intestine.

Bacterial RNA polymerase (RNAP) constitutes the central enzyme for gene expression that transcribes DNA to RNA in a highly regulated process and remains pivotal for the survival of the bacteria (31). The potential of bacterial RNA polymerase as a therapeutic target stems from its indispensable biological function, highly conserved nature, and sufficient divergence from its eukaryotic counterpart rendering toxicity to be a rare event (11). Indeed, a plethora of literature reports the emergence of RNA polymerase as a promising target for PNAs against Gram-positive and Gram-negative pathogens of clinical importance (11, 32 – 34). Our designed peptide-PNA conjugate was aimed at inhibiting the expression of the *rpoA* gene in *C. difficile*. Using RT-PCR, we confirmed that the designed *rpoA*-TAT conjugate specifically inhibited the expression of the *rpoA* gene by 78.2% at 0.5× MIC. Experimental, epidemiological, and clinical evidence convincingly shows *C. difficile* toxins, toxin A (*tcdA*) and toxin B (*tcdB*), are the key determinants of disease pathogenesis. Both toxins A and B can catalyze glycosylation and thereby inactivate Rho-GTPases leading to disorganization of the cellular cytoskeleton and apoptosis of intestinal epithelial cells (35). The inhibition of *rpoA* gene expression by the *rpoA*-TAT conjugate resulted in a dose-dependent inhibition of the expression of *tcdA* and *tcdB* by 71.2% and 74.5% at 0.5× MIC, respectively. Thus, targeting *rpoA* gene expression indirectly downregulates the expression of the toxin genes, which can confer an added therapeutic advantage to the use of our peptide-PNA conjugate. We further validated the inhibition of toxin production using an ELISA, which confirmed a 55.2% inhibition of toxin production when *C. difficile* 630 was exposed to 0.5× MIC of the *rpoA*-TAT conjugate.

Bacteria have evolved to survive in adverse environmental conditions; one mechanism certain bacteria like *C. difficile* use to survive in such conditions involves transitioning from a vegetative cell to a dormant and environmentally resistant cell type called a spore (36). Efficient transmission of *C. difficile* in the environment is facilitated by these aerotolerant and highly resistant spores (37). Indeed, a major source of infection in the hospital environment remains the shedding of *C. difficile* spores by infected patients (38). Of the standard-of-care antibiotics used to treat CDI, vancomycin can inhibit the growth of vegetative cells but cannot inhibit spore formation by *C. difficile*. Fidaxomicin is the only standard-of-care antibiotic for CDI that exhibits the potential to inhibit spore formation (38). Our qPCR data revealed that inhibition of the *rpoA* gene by the *rpoA*-TAT conjugate also downregulated the expression of *spoOA* (68.5% reduction at 0.5× MIC) that encodes for the transcription factor stage 0 sporulation protein involved, which is responsible for the initiation of sporulation in *C. difficile* (39). In fact, previous literature shows that a *spoOA* mutant results in an asporogenous phenotype in *C. difficile* (40). Furthermore, a spore formation inhibition assay validated our qPCR data where we found that *C. difficile* failed to form spores when exposed to 0.5× MIC of the peptide-PNA conjugate. We noted a 3.44-log reduction (99.9% reduction) in *C. difficile* spore count for both fidaxomicin and the *rpoA*-TAT conjugate when compared to the negative control.

The undisputed association between antibacterial therapy and infection caused by *C. difficile* entails a targeted therapy that will interfere only with *C. difficile* growth and expression of its virulence factors. Bacterial RNAP has been found to vary greatly, even among bacteria, as the regulatory networks keep on adjusting the gene expression process to environmental cues (41). Indeed, the novel macrolide antibiotic fidaxomicin, which does not harbor potent activity against crucial commensals like *Bacteroides*, targets the clostridial RNAP (42). An important finding of our study was that the *rpoA*-TAT conjugate did not inhibit the growth of commensal microflora species that we evaluated.

Despite the increasing clinical significance of *C. difficile*, there is a lack of effective genetic tools. Here, we designed a carrier CPP-peptide conjugate that can target *C. difficile* with the added benefit of being able to inhibit the expression of virulence factors by the pathogen. In addition to the proposed novel treatment for *C. difficile* infections, we also propose a novel approach to manipulate gene expression in *C. difficile* without the need for established genetic tools.

## MATERIALS AND METHODS

### Bacterial strains, reagents, and kits

The bacterial isolates were obtained from the American Type Culture Collection (ATCC), the CDC, and the Biodefense and Emerging Infections Research Resources Repository (BEI Resources, Manassas, VA). *C. difficile* strains were grown in Mueller-Hilton broth II (Beckton, Dickinson, and Company, MD) or supplemented brain heart infusion agar plates (BHIS, Beckton, Dickinson, and Company, MD) at 37°C in an anaerobic chamber (Coy Laboratory Products Inc., Grass Lake, MI). Vancomycin hydrochloride (Sigma-Aldrich, St. Louis, MO) and fidaxomicin (Cayman Chemicals, Ann Arbor, MI) were purchased from commercial vendors. *TRI*zol Max Bacterial RNA Isolation Kit, Turbo DNA-free Kit, SuperScript III First-Strand Synthesis Kit (Invitrogen, Carlsbad, CA), and iTaq Universal SYBR Green One-Step Kit were purchased from commercial vendors (Bio-Rad Laboratories, Inc., Hercules, CA).

### Peptide nucleic acids and cell penetrating peptide

A PNA oligomer targeting the *rpoA* gene in *C. difficile* 630 was designed to be complementary to a 12-nucleotide target sequence that included the start codon and nine nucleotides upstream of the start codon including the ribosomal binding site. The CPPs [TAT, (RXR)_4_XB, (RFR)_4_XB, K8, and (KFF)_3_] were covalently conjugated to the designed PNA oligomer. These PNA constructs were synthesized by PNA Bio Inc. (Thousand Oaks, CA). Free CPP was synthesized by GenScript (Piscataway, NJ).

### *In vitro* anticlostridial activity of the CPP and CPP-PNA conjugates

The susceptibility of *C. difficile* isolates to the CPP-PNA conjugates or to the CPP alone was determined *in vitro* as described before with slight modifications (43 – 45). Briefly, the CPP-PNA conjugates, the CPP alone, and the antibiotics vancomycin and fidaxomicin were added in triplicate to the first row of ultra-low adhesion 96-well plates (Corning Inc., Corning, NY). A suspension of *C. difficile* equivalent to 0.5 McFarland solution was added to Mueller-Hilton II broth, which was then added to the ultra-low adhesion plate and then serially diluted. The plates were incubated anaerobically for 48 hours at 37°C. The minimum inhibitory concentration was defined as the lowest concentration of the CPP-PNA conjugate that could inhibit bacterial growth when observed visually.

Aliquots from wells of the 96-well plate that did not show bacterial growth were further plated on BHIS agar plates to determine the minimum bactericidal concentration for the PNA construct. The highest dilution of the PNA construct that did not show bacterial growth was defined as its MBC (45). The MIC and MBC experiments were repeated twice.

### Killing kinetics of *rpoA*-TAT

The killing kinetics of the PNA construct was determined as described previously (45 – 47). Briefly, an overnight culture of *C. difficile* 630 was diluted 1:50 into fresh Mueller Hilton II broth (~10^5^ CFU/mL) and added in triplicate to low adhesion microcentrifuge tubes containing either sterile water, the *rpoA*-TAT construct (1× MIC), or the positive controls vancomycin or fidaxomicin (1× MIC). The number of viable bacteria in each treatment group was determined by taking aliquots at 0, 4, 8, 12, 16, and 24 hours; these aliquots were then serially diluted and plated on BHIS agar plates. The plates were incubated anaerobically, and the number of colony-forming units for each treatment group was recorded the following day. The experiment was repeated twice.

### Evaluation of inoculum effect on *C. difficile* susceptibility to PNA construct

The *in vitro* efficacy of the PNA constructs against a high inoculum size of *C. difficile* 630 was determined. Briefly, the PNA constructs and control antibiotics, at a concentration of 1× MIC, were added in triplicate to low adhesion microcentrifuge tubes (Corning Inc., Corning, NY). A high inoculum of *C. difficile* suspension equivalent to ~5 × 10^6^ and ~5 × 10^7^ cells was added to the tubes, which were incubated anaerobically at 37°C. The number of viable bacteria in each treatment group was determined by serial dilution and plating on BHIS agar plates at 8 hours and 24 hours post-incubation, respectively. The effect of a high inoculum on the activity of the PNA construct was repeated twice.

### Evaluation of the effect of pH on the antibacterial activity of the PNA construct

The susceptibility of *C. difficile* (ATCC 630 and ATCC BAA 1870) to the *rpoA*-TAT conjugate was evaluated over a pH range of 6 to 9 using the broth microdilution method (43, 46 – 48). The desired pH for susceptibility testing was attained by the addition of either 0.1 N HCL or 1 N NaOH to the media. The experiment was repeated twice.

### Evaluation of the effect of the *rpoA*-TAT conjugate on gene expression in *C. difficile*

The antisense effect of the *rpoA*-TAT conjugate was evaluated using a real-time quantitative reverse-transcriptase polymerase chain reaction (RT-PCR) assay (11, 12). Briefly, an overnight culture of *C. difficile* ATCC 630 was diluted in fresh Mueller-Hilton broth II and added in triplicate to low-adhesion microcentrifuge tubes. Prior to adding bacteria to the tubes, the PNA-TAT construct was added in the following concentrations: 0.125× MIC (1 µM), 0.25× MIC (2 µM), and 0.5× MIC (4 µM), respectively. The tubes were incubated anaerobically at 37°C for 12 hours following which the bacterial pellet in each treatment group was collected by centrifugation at 10,000 × *g* for 10 minutes. Bacteria were subsequently lysed with recombinant lysostaphin (100 µg/mL) in Tris-EDTA (TE) buffer (1 mol/L Tris, 0.5 mol/L ethylenediaminetetraacetate pH 8, nuclease-free H_2_0), and total RNA was extracted using the *TRI*zol Max Bacterial Isolation Kit as per the manufacturer’s instructions. Turbo DNAse was used to remove genomic DNA contamination from each sample as per the protocol. cDNA was synthesized using the SuperScript III First-Strand Synthesis Kit as per the manufacturer’s instructions. The relative quantification of cDNA for each treatment group was determined using the iTaq Universal SYBR Green One-Step Kit and CFX96 Real-Time PCR Detection System per the manufacturer’s instructions.

The sequences of the primers used are shown in Table S3. The constitutively expressed *tpi* gene was used as an internal control. Real-time RT-PCR results were analyzed using the 2^-ΔΔCT^ method.

### Evaluation of toxin inhibition by the *rpoA*-TAT conjugate

To evaluate the inhibition of *C. difficile* toxin production (45, 47 – 49) by the *rpoA*-TAT conjugate, *C. difficile* 630 (~10^6^ CFU/mL) was added to low adhesion microcentrifuge tubes containing either water (negative control), *rpoA*-TAT (0.125× MIC, 0.25× MIC, and 0.5× MIC), or the standard-of-care antibiotics vancomycin and fidaxomicin (0.125× MIC, 0.25× MIC, and 0.5× MIC). After the incubation period, the bacterial count in each treatment group was determined by serial dilution and plating. Toxin content in the supernatant of each treatment group was determined using an enzyme-linked immunosorbent assay (ELISA, tgcBIOMICS) kit, as per the manufacturer’s protocol.

### Spore formation inhibition by the *rpoA*-TAT conjugate

To evaluate the inhibition of spore formation by the *rpoA*-TAT conjugate, *C. difficile* ATCC 630 (~10^6^ CFU/mL) was added to low adhesion microcentrifuge tubes containing either water (negative control), *rpoA*-TAT (0.5× MIC), or the standard-of-care antibiotics vancomycin and fidaxomicin (0.5× MIC) and incubated anaerobically for 12 hours. After the incubation period, the total bacterial count for each treatment group was determined by serial dilution and plating on BHIS agar plates supplemented with 0.1% taurocholic acid. To determine the presence of spores, the remaining bacterial culture from each treatment group was heated at 65°C for 30 minutes to ensure all vegetative cells were killed. The bacterial culture from each treatment group was then serially diluted and plated on BHIS agar plates supplemented with 0.1% taurocholic acid. The plates were incubated overnight, and the CFU counts for each treatment group were determined the following day.

### *In vitro* susceptibility of gut microflora strains to the CPP-PNA conjugate

The susceptibility of gut microflora strains to the *rpoA*-TAT conjugate was determined using the broth microdilution assay, as described above. Briefly, bacterial suspensions of *Bifidobacterium* sp. and *Bacteroides* sp., in supplemented BHIS broth, and Lactobacilli strains, in MRS broth, were added to ultra-low adhesion 96-well plates and serially diluted. The MIC was recorded after 48 hours.

## Data Availability

The authors confirm that the data supporting the findings of this study are available within the article and/or its supplementary material.

## SUPPLEMENTAL MATERIAL

Supplemental tables spectrum.01755-23-s0001.pdf Tables S1 to S4.

ASM does not own the copyrights to Supplemental Material that may be linked to, or accessed through, an article. The authors have granted ASM a non-exclusive, world-wide license to publish the Supplemental Material files. Please contact the corresponding author directly for reuse.
