# Supplementary material for: Antisense inhibition of RNA polymerase α subunit of Clostridioides difficile
Source: Microbiol Spectr. 2023 Sep 29;11(5):e01755-23. doi: 10.1128/spectrum.01755-23 (PMC10581251; doi:10.1128/spectrum.01755-23)
Supplement: Supplemental tables — Tables S1 to S4. [file spectrum.01755-23-s0001.pdf]

Table S1: Sequence alignment of DNA-directed RNA polymerase α subunit (*rpoA*) gene among *C. difficile* isolates:

Organism	Locus tag	Identity	Sequence (5'-3')
Clostridioides difficile ATCC 630	CD630_00980	100%	TTAAGGAGGGTTTTGTCCATGATAGAAATAGA
Clostridioides difficile R20291	CDIF27147_00173	100%	TTAAGGAGGGTTTTGTCCATGATAGAAATAGA
Clostridioides difficile ATCC 9689	CDIF1296T_00168	100%	TTAAGGAGGGTTTTGTCCATGATAGAAATAGA
Clostridioides difficile strain DSM 27638	CDIF27638_RS00835	100%	TTAAGGAGGGTTTTGTCCATGATAGAAATAGA
Clostridioides difficile strain DSM 27639	CDIF27639_RS00805	100%	TTAAGGAGGGTTTTGTCCATGATAGAAATAGA
Clostridioides difficile strain DSM 27640	CDIF27640_RS00835	100%	TTAAGGAGGGTTTTGTCCATGATAGAAATAGA
Clostridioides difficile strain MHS-156	KK110_RS00825	100%	TTAAGGAGGGTTTTGTCCATGATAGAAATAGA

Table S2: Strains of *C. difficile* used in the study

**Strains**	**Comments**
C. difficile ATCC 630	Toxigenic strain; presence of *tcdA,* and *tcdB* genes
C. difficile ATCC BAA 1870	Toxigenic strain; presence of *cdtB, tcdA,* and *tcdB* genes
C. difficile ATCC 43255	Toxigenic strain; presence of *tcdA,* and *tcdB* genes
C. difficile CDC 1071	Toxigenic strain; presence of *cdtB, tcdA,* and *tcdB* genes
C. difficile CDC 1079	Toxigenic strain; presence of *tcdA,* and *tcdB* genes
C. difficile CDC 1082	Toxigenic strain; presence of *tcdA,* and *tcdB* genes

Table S3: Sequence of primers used in the study:

Gene	Primers
rpoA	GCTCTATCACAGGTGCAGATATAA
	TCAGCAGAAACATAACCTCTACC
tcdA	ACTAGACGAACATGACCCATTAC
	ACTTCAGCACCTATTCCAACAA
tcdB	CATGTTAGACGAAGAAGTTCAAAGT
	CTAGTGGTGATGCCTCCATATC
spoOA	CTAGACCAGTTCAAGAGACTCAAA
	GAGCTGGTACTCCTATTTCATGT
tpi	AGGTTTAACACTTCCACCGTATT
	TGAGCCAATCTGGGCTATTG

Table S4: Strains of commensal microflora used in the study

Strains	Source
Lactobacillus brevis ATCC 14869	Isolated from feces
Lactobacillus gasseri ATCC 19992	Isolated from feces

Lactobacillus rhamnosus ATCC 53103	Isolated from feces
Bacteroides fragilis HM 714	Isolated from feces
Bifidobacterium longum subsp. *longum* HM 846	Isolated from feces
